# Evaluation of the Critical Care Team Training and Clinical Excellence (TTrACE) Academy

**DOI:** 10.3390/healthcare14152421

**Published:** 2026-08-06

**Authors:** Kari Carhart, Natalie Weiser, Ryan Brydges, Robyn Davies, Donna Romano, Sabrina Deutsch Salamon, Karlie-Carmen DeAngelis, Nichelle Benny Gerard, Sonya Canzian, Jane Topolovec-Vranic

**Affiliations:** 1 Unity Health Toronto, 30 Bond St., Toronto, ON M5B 1W8, Canada; kcarhart@haltonhealthcare.com (K.C.); natalie.weiser@unityhealth.to (N.W.); ryan.brydges@utoronto.ca (R.B.); robyn.davies@unityhealth.to (R.D.); donna.romano@unityhealth.to (D.R.); karli.deangelis@unityhealth.to (K.-C.D.); nichelle.bennygerard2@unityhealth.to (N.B.G.); sonya.canzian@unityhealth.to (S.C.); 2 Li Ka Shing Knowledge Institute, 209 Victoria St., Toronto, ON M5B 1A6, Canada; 3 Institute of Health Policy, Management and Evaluation, University of Toronto, 155 College Street, Toronto, ON M5T 3M6, Canada; 4 Department of Physical Therapy, Faculty of Medicine, University of Toronto, 500 University Avenue, Toronto, ON M5G 1V7, Canada; 5 Lawrence Bloomberg Faculty of Nursing, University of Toronto, 155 College Street, Toronto, ON M5T 3M6, Canada; 6 School of Administrative Studies, York University, 4700 Keele St., Toronto, ON M3J 1P3, Canada; sdeutsch@yorku.ca; 7 School of Human Resource Management, York University, 4700 Keele St., Toronto, ON M3J 1P3, Canada

**Keywords:** workforce well-being, highly functional teams, teamwork, critical care, health professional education, interprofessional education, program evaluation

## Abstract

**Purpose:** Sustaining high-functioning interprofessional teamwork in intensive care settings is essential for patient safety, workforce well-being, and reliable care delivery. However, evidence regarding the role of structured team training interventions in already high-performing critical care teams remains limited. The purpose of this study was to assess the impact of the Team Training and Clinical Excellence Academy (TTrACE), a structured interprofessional team training program, on collaboration, psychological safety, need satisfaction, and team effectiveness in a critical care setting. **Methods:** We conducted a prospective pre–post evaluation of an interprofessional critical care team training program in a medical/surgical ICU at a large academic hospital. The evaluation used previously published measures of collaborative environments and psychological need satisfaction, along with exploratory team-functioning items assessing shared mental models, psychological safety and communication, and perceived team effectiveness. **Results:** Of the 35 TTrACE enrollees, 28 (80%) consented to participate in the evaluation study. Baseline scores across collaboration, psychological need satisfaction, and team functioning were high and remained stable at one-month follow-up, suggesting preservation of strong team functioning over time. Descriptive improvements were observed in perceived teamness and shared mental models. Most respondents reported having an opportunity to implement TTrACE learnings in practice (11/13, 84.6%; 1 missing response). All respondents who answered the recommendation item indicated they were likely or very likely to recommend TTrACE to colleagues. **Conclusions:** In high-performing critical care environments, structured interprofessional training may contribute less to large measurable performance gains and more to reinforcing and sustaining relational, communicative, and psychological processes essential for safe patient care. These findings suggest that TTrACE may support the maintenance of high-functioning team environments in critical care, while future longitudinal and comparative studies are needed to examine longer-term impacts on team functioning, workforce well-being, and patient safety outcomes.

## 1. Introduction

Intensive care units (ICUs) are complex, high-risk clinical environments that require rapid decision-making, effective communication, and coordinated action across multiple professional groups. Interprofessional collaboration and strong team functioning in critical care settings are fundamental to providing excellent patient care. High-functioning teams in the ICU improve situation awareness, enable rapid and accurate decision-making, reduce errors, and increase consistent use of protocols (e.g., ventilator and sepsis bundles), all of which translate into measurable improvements in care processes and, in some studies, reductions in morbidity and mortality [1].

Interventions aimed at strengthening team functioning have demonstrated positive outcomes for both staff and patients [2,3]. Interprofessional collaboration enhancements such as structured interdisciplinary rounds, regular interprofessional conferences, team training, and targeted interprofessional education have been shown to improve both care processes and patient outcomes in critical care settings. For example, implementation of interprofessional structured rounds (often supported by checklists) enhanced communication, clarified task assignments, and reduced care omissions, leading to improvements in process measures such as adherence to evidence-based practices and reductions in delays of critical interventions [4]. The use of structured rounding checklists during interprofessional rounds was associated with improved teamwork and communication [5] and has been linked to reduced ICU and hospital length of stay, lower mortality, and decreased rates of device-associated infections compared with nonstructured practice [6,7]. Interprofessional education and team training in critical care have been shown to improve teamwork behaviours, care processes, and patient safety outcomes in highly complex clinical environments. Interprofessional and simulation-based team training enhances communication, role clarity, shared mental models, leadership, and psychological safety, enabling clinicians to anticipate needs, speak up, and coordinate care more effectively during both routine and high-acuity situations [3,8,9]. These improvements are associated with better adherence to evidence-based practices, more reliable implementation of care bundles, improved handovers, and enhanced recognition and response to clinical deterioration [2,10]. Importantly, several systematic reviews and controlled studies report that strengthened team functioning and care reliability translate into reductions in adverse events and preventable complications and, in some settings, shorter ICU and hospital length of stay [2,3,10,11]. While effects on mortality are variable, the strongest and most consistent evidence supports improvements in safety, efficiency, and care reliability, outcomes that are particularly critical in intensive care settings, where failures in teamwork can have significant consequences [3,11,12].

Despite this evidence, several gaps remain. Although prior research demonstrates that team training interventions can improve teamwork and patient safety outcomes, most studies have focused on settings with identifiable deficits in communication or collaboration [2,3,8,9]. Less is known about the role of structured interprofessional training in sustaining performance with already high-functioning critical care teams. This is an important gap because high-performing teams may also require ongoing reinforcement to maintain psychological safety, trust, shared accountability, and collaborative reliability over time.

To address this gap, the Team Training and Clinical Excellence Academy (TTrACE), a unique evidence-informed, structured interprofessional team training program, was developed within the critical care program of a large urban academic health sciences centre in Toronto, Canada. TTrACE aimed to support a positive learning and work environment that strengthened relationships, connections, and accountability for delivering safe, high-quality patient- and family-partnered care experiences [13,14].

We aimed to evaluate this pilot implementation of the TTrACE program using multiple data sources. In addition to collecting baseline and post-implementation data on attendees’ perceptions of important team functions (e.g., team effectiveness, collaboration, psychological safety, and need satisfaction), we sought their feedback and suggestions for program refinement. We hypothesized that participation in TTrACE would reinforce and sustain high levels of collaboration, psychological safety, and team effectiveness among ICU professionals.

The TTrACE Academy was developed in response to a demonstrated need for formalized team training identified through staff engagement survey findings. The Medical Surgical Intensive Care Unit (MSICU) was selected as the pilot site due to its demonstrated readiness to participate and strong leadership support. Initial curriculum development was informed by a targeted literature review focusing on team function, high-performing teams, team performance, and teamwork within healthcare contexts, as well as an analysis of institutional safety reports. Qualitative insights into team member perspectives were further obtained through in-person huddles using structured, scripted questions. Curriculum content was subsequently developed through the synthesis of evidence-based characteristics of high-functioning teams and locally identified practices. Program development, implementation, and evaluation were guided by a formal governance structure comprising an advisory group, including patient and family partners, operational working groups, and an executive committee.

The curriculum was theoretically informed by the Canadian Interprofessional Health Collaborative (IPC) Competency Framework [13], which identifies core domains for effective interprofessional collaborative practice, including interprofessional communication, role clarification, team functioning, collaborative leadership, interprofessional conflict resolution, and patient/client/family/community-centred care. These domains aligned with the intended focus of TTrACE on strengthening communication, psychological safety, trust, shared accountability, collaborative decision-making, and patient- and family-partnered care within critical care teams. The program was also informed by the Institute for Healthcare Improvement Joy in Work Framework [14], which supported attention to workforce well-being, meaning, connection, and the creation of conditions that enable staff to provide high-quality care.

Delivered in person over two consecutive days, the TTrACE program comprised six core courses: High Functioning Teams; Emotional Intelligence; Psychological Safety and Trust; Advanced Communication; Health Equity; and Relational Ethics and Having Difficult Conversations. Content delivery was multimodal and included didactic sessions, interactive team-based activities, simulations, and discussion panels. Integrated throughout the program were videos and verbal testimonials provided by patient and family partners who participated in curriculum development.

Throughout the training, participants were encouraged to engage in candid discussions about the healthcare experience. As a culminating activity, each group of participants developed a team charter, building on the concepts explored during the two-day program.

## 2. Methods

### 2.1. Study Design

We conducted a prospective, single-group, pre–post program evaluation study using quantitative surveys and qualitative open-ended feedback. The evaluation focused on participants’ perceptions of interprofessional collaboration, psychological safety, motivational climate, team functioning, and early application of learning in practice. Participants were recruited by email and completed electronic surveys (via SurveyMonkey Inc. version 2024; San Mateo, CA, USA; www.surveymonkey.com) at baseline (prior to program participation) and at 1 month post-enrollment. Research ethics approval was granted by the Research Ethics Board of the study site (REB 23-099).

### 2.2. Setting and Participants

The TTrACE program was implemented in the MSICU of a large academic health sciences centre in Toronto, Canada. Participation in the program was offered to the entire interprofessional unit team (i.e., nursing, medicine, and health professions), with 35 staff enrolling across two offerings in fall 2023 and winter 2024. All enrollees were subsequently invited to participate in the evaluation study that used a pragmatic census sampling approach: an email was sent to TTrACE enrollees by an independent research coordinator with a letter of information and a link to access the consent form. Surveys were coded with a de-identified study ID. Participation in both the TTrACE program and the evaluation study was voluntary. Eligible participants included all healthcare professionals enrolled in one of the two TTrACE cohorts [14,15].

### 2.3. Data Collection

#### 2.3.1. Procedure

TTrACE enrollees who consented to participate in the evaluation were sent an electronic link to complete the study surveys via SurveyMonkey prior to the start of the program (baseline) and again 1 month post-program (follow-up). The following data were collected at each time point:

1. Demographic survey (baseline only): Participants were asked to indicate their profession, how long they had been practicing in their profession, how long they had been working at the study hospital and their practice unit, gender (male/female), age, and highest level of education attained.
2. Assessment for Collaborative Environments (ACE-15): The ACE-15 is a previously developed 15-item self-report measure designed to assess perceptions of interprofessional “teamness” in clinical learning and practice environments [16]. The measure was developed for use across a range of clinical settings and health professionals to provide a rapid assessment of teamwork qualities in interprofessional environments. Items are scored on a 4-point Likert scale, with three negatively worded items reverse-scored. Summed scores range from 15 to 60, with higher mean scores indicating higher perceived teamness and lower variability indicating less internal disagreement in perceptions of teamwork [16].
3. Basic Psychological Need Satisfaction and Frustration Scale (BPNSFS): The BPNSFS is a previously developed and psychometrically evaluated 24-item self-report measure grounded in self-determination theory [17]. The scale assesses the extent to which individuals experience satisfaction and frustration of the three basic psychological needs of autonomy, competence, and relatedness [1]. The BPNSFS includes six subscales assessing autonomy satisfaction, autonomy frustration, competence satisfaction, competence frustration, relatedness satisfaction, and relatedness frustration, with items rated on a Likert scale and scores typically averaged or summed. A higher mean score on satisfaction subscales reflects greater perceived need fulfillment, whereas a higher mean score on frustration subscales reflects greater perceived need undermining [17].
4. Exploratory team functioning items: Team functioning was assessed using a set of 20 exploratory items assembled a priori to align with the objectives and curriculum domains of TTrACE. These items were organized into three domains: shared mental models (items 1–5), psychological safety and communication (items 6–12) [18], and perceived team effectiveness (items 13–20). Items 6–12 correspond to Edmondson’s 7-item Psychological Safety Scale, which assesses perceptions of interpersonal risk-taking and inclusion within teams [18]. Shared mental models were assessed using items reflecting team members’ shared understanding of goals, roles, and task processes, consistent with established definitions of team mental models as overlapping cognitive representations of task and team functioning [19]. The remaining items (13–20) assessed perceived team effectiveness and were drawn from previously validated team climate/team functioning measures [20]. These items were assembled for the purposes of this pilot evaluation and were not validated as a single composite instrument. Results are presented descriptively by domain and interpreted as exploratory.
5. Follow-up survey on learning implementation (follow-up only): This 5-item survey assessed whether participants endorsed implementing their learnings from TTrACE into their practice in the prior two weeks, whether participating in the program influenced their interactions with their interprofessional team, what TTrACE content they had found most and least valuable, whether they had suggestions for additional modules, and whether they would recommend participating in TTrACE to their colleagues.

The selected instruments captured complementary dimensions of interprofessional team functioning, including collaboration, psychological safety, motivational climate, and perceived effectiveness.

#### 2.3.2. Data Analyses

Demographic data were summarized using descriptive statistics. Due to the small follow-up sample size and the inability to pair participant responses across time points, inferential pre–post statistical analyses were not considered appropriate. Accordingly, findings are presented descriptively (for example, means [SD] and ranges) to characterize trends and patterns over time. Open-ended responses were analyzed using inductive thematic analysis to identify recurring patterns. Two members of the research team independently reviewed responses, generated preliminary codes, and collaboratively developed overarching themes through iterative discussion.

## 3. Results

### 3.1. Characteristics of Participants

Twenty-eight participants completed the baseline surveys, and 14 responded to the follow-up assessment at one month post-TTrACE completion.

Baseline participant characteristics are summarized in Table 1: participants were predominantly female (82%), with 68% reporting ≥ 9 years in their profession. Respondents represented multiple professional groups, most commonly health disciplines (43%) and nursing (32%). The largest age category was 20–29 years (43%), and most participants reported an undergraduate (57%) or master’s degree (32%) as their highest level of education. Given that only 13/28 participants provided full data sets, which were anonymized and do not permit paired analyses, we did not use any inferential statistics in our reporting below.

### 3.2. ACE-15 Survey

At baseline, 28 participants completed the survey, with ACE-15 totals ranging from 34 to 59 (M = 44.4, SD = 4.9). Core teamwork processes were frequently reported (e.g., shared goal-setting, autonomy once patient goals were clear, and a culture of continuous learning), whereas comparatively fewer respondents selected response options reflecting routine performance evaluation, trust-building, and psychological safety to raise concerns. Fourteen participants completed the ACE-15 follow-up survey, producing scores that ranged from 39 to 53 (M = 47.0, SD = 4.3), suggesting relative stability at 1 month post-program completion. Although baseline perceptions of teamness were already high, follow-up findings suggested sustained collaborative functioning, with descriptive improvements in perceived team cohesion and shared understanding of team processes.

### 3.3. BPNSFS Survey

Participants’ BPNSFS scores were high at baseline and remained similar post-participation (Table 2), with the highest mean scores observed for competence satisfaction and relatedness satisfaction. Need frustration scores were low to moderate and also remained stable, with the lowest means observed for relatedness frustration.

### 3.4. Exploratory Team Functioning Items

Exploratory team functioning domain scores were high at baseline and remained high post-participation (Table 3). Scores remained descriptively similar across time points. The largest descriptive increase was observed in shared mental models, suggesting that TTrACE may have strengthened team members’ shared understanding of goals, communication processes, and collaborative roles.

### 3.5. Integrated Patterns Across Team Functioning Measures

High baseline scores across measures suggest participants were functioning within an already mature and collaborative team environment, potentially limiting the magnitude of measurable post-intervention change. Across the ACE-15, BPNSFS, and exploratory team-functioning items, scores remained consistently high at follow-up, suggesting preservation of strong interprofessional team functioning over time. Sustained psychological safety and communication may have supported participants’ ability to speak up, share concerns, and engage in collaborative dialogue, while the descriptive increase in shared mental models suggests strengthened common understanding of team goals, roles, and processes. These patterns suggest that psychological safety, shared mental models, and perceived team effectiveness may reinforce one another within highly functional teams. In this context, the sustained maintenance of high team-functioning scores may itself represent a meaningful outcome, particularly in a complex critical care environment where preserving psychological safety, communication, and shared understanding is essential for reliable team performance.

### 3.6. Post-Participation General Survey (n = 14)

Most respondents reported having an opportunity to implement Academy learnings in practice (11/13, 84.6%). All respondents who answered the recommendation item indicated they were likely or very likely to recommend the Academy to colleagues (13/13; 69.2% “very likely,” 30.8% “likely”).

Open-ended evaluation responses identified several recurring themes spanning participants’ expectations, perceived value, and recommendations for refinement.

Anticipated learning needs emphasized team functioning, communication, equity, and self-awareness. Prior to the Academy, participants described learning needs focused on strengthening team functioning and collaboration, developing practical communication strategies for team and family-facing interactions, and building equity-oriented awareness and advocacy in care. Participants also highlighted personal and professional development goals, including increased self-awareness and the role of emotions in teamwork.

Post-participation, most participants valued elements focused on communication, teamwork, and patient and family perspectives. Participants frequently described communication as highly valuable, including improving communication with team members and strengthening understanding of the patient and family experience to inform bedside care and overall patient experience. Patient and family involvement was repeatedly described as impactful (for example, “Fantastic to hear directly from patients and families” and “Having the panel of patients and their SDM to share their story was nice to hear their perspective”). Respondents also emphasized teamwork and collaboration, including the value of co-developing a shared team philosophy and recognizing diverse expertise across roles.

Participants described early application of skills, with workload as a key implementation constraint. Post-program reflections indicated that several participants applied Academy learning in day-to-day practice, including communication strategies in team interactions, shared decision-making, family meetings, debriefs, and using emotional intelligence to manage emotions in difficult discussions. Some participants noted difficulty identifying specific examples or fully implementing strategies due to workload constraints.

Suggested refinements centred on increasing practical application, time for discussion, and supportive materials. Across comments, participants requested more practical strategies and case-based activities (for example, “I would have appreciated more practical strategies” and “More time for case work”). Respondents also suggested additional time for patient and family panels and Q&A and clearer up-front structure (for example, agenda or context). Recommendations for learning supports included more written resources (learning guides, cue cards), expanded multimedia and interactive elements (videos, scenarios), and added content on conflict resolution, ethics, equity in practice, and patient and family advocacy.

## 4. Discussion

This study evaluated the impact of a structured interprofessional team training program (TTrACE) in a critical care setting on collaboration, psychological safety, and team effectiveness. Findings demonstrated high levels of team functioning at baseline that were sustained at one month post-program across all measured domains, including collaborative environments, basic psychological need satisfaction, and overall team effectiveness. Our findings suggest that, within highly functioning ICU teams, structured interprofessional training may serve a maintenance and reinforcement function rather than a corrective one. In complex, high-risk environments where psychological safety and collaborative reliability are essential, preserving strong team dynamics may itself represent a meaningful organizational outcome. These findings align with broader theories of organizational resilience and high reliability in healthcare, where sustained team cohesion, trust, and communication are considered essential for maintaining safe performance under conditions of complexity and uncertainty [21,22,23].

The consistently high scores observed both pre- and post-intervention likely reflect a mature team environment characterized by strong psychological safety, trust, and shared understanding of roles and goals. Over two-thirds of the participants had been in their profession for over 9 years, with 54% working on their unit for over 3 years. This aligns with existing theory suggesting that psychological safety and team effectiveness are dynamic constructs that require ongoing reinforcement to be maintained over time [18]. 

The sustainment of psychological safety is particularly important in intensive care settings, where clinicians must continuously speak up, ask questions, raise concerns, and adapt communication in response to rapidly changing patient conditions [18]. In this context, psychological safety is not only a marker of positive team climate but also a practical condition for error prevention, escalation of concerns, and shared clinical decision-making [18]. The stability of psychological safety and communication scores at follow-up may therefore reflect preservation of an important relational foundation for safe and reliable critical care [18].

The training program may have contributed to reinforcing shared mental models, communication norms, and mutual respect among team members, all of which are foundational to effective interprofessional collaboration. Prior research has demonstrated that team training interventions, including simulation and Crew Resource Management approaches, enhance non-technical skills such as communication, leadership, and coordination [3,24]. Our findings extend this work by suggesting that such interventions are also valuable in preserving high baseline performance, an outcome that is less frequently reported in the literature.

TTrACE may also be understood as a cultural intervention rather than solely an educational program. Beyond delivering content on teamwork and communication, the program created structured time for interprofessional reflection, dialogue, patient and family partner perspectives, and development of a shared team charter. These activities may have reinforced shared values, collective identity, and expectations for how team members communicate, collaborate, and support one another in practice. In mature clinical teams, this type of structured training may function as a mechanism of cultural reinforcement by making implicit norms around trust, psychological safety, and shared accountability more visible and actionable.

Much of the existing evidence on interprofessional team training focuses on improvements from lower baseline levels of team functioning, often demonstrating gains in teamwork behaviours and patient safety outcomes following intervention [2]. In contrast, our findings highlight a different but equally important effect: the sustainment of high levels of collaboration, trust, and psychological safety over time. This is particularly relevant in critical care settings, where maintaining consistently high team performance is essential to patient safety and quality of care [2,10,25].

Additionally, the sustained high scores on the BPNSFS suggested that the work environment continued to support autonomy, competence, and relatedness, which are key drivers of motivation and well-being according to self-determination theory [17]. This reinforces the idea that team training interventions may contribute not only to team processes but also to positive work environment conditions.

## 5. Theoretical Implications

Theoretically, these findings extend traditional improvement-oriented models of team training by suggesting that structured interprofessional education may also support the sustainment of excellence in mature clinical teams. In this study, high baseline scores across collaboration, psychological safety, need satisfaction, and team effectiveness suggest that participants were already working within a mature and collaborative team environment. In this context, the sustained high scores observed at follow-up may reflect the role of TTrACE in maintaining key relational and collaborative conditions required for effective critical care teamwork.

The findings also reinforce the theoretical importance of psychological safety and shared mental models in high-acuity clinical environments. Psychological safety supports speaking up, asking questions, raising concerns, and engaging in learning behaviours, while shared mental models enable team members to develop a common understanding of goals, roles, communication processes, and task demands [18,19]. The descriptive improvement observed in shared mental models suggests that TTrACE may have strengthened shared understanding of team processes, even in a setting where overall team functioning was already rated highly.

## 6. Implications for Practice

These findings have important implications for health system leaders and critical care teams. First, they support the integration of structured interprofessional team training as an ongoing component of workforce development, even in high-performing environments. Second, they highlight the importance of focusing on team sustainment strategies, not just performance improvement. Embedding regular team training may help preserve psychological safety, trust, and collaboration, which are essential for effective team functioning and staff well-being [3,18,24,26].

For critical care leaders, these findings suggest that team training could be implemented in response to teamwork challenges and proactively to reinforce shared expectations, communication norms, role clarity, and collaborative decision-making. This is particularly relevant in ICU environments, where high workload, clinical complexity, staff turnover, and rapid decision-making can place ongoing pressure on team functioning. Maintaining structured opportunities for interprofessional reflection and shared learning may help sustain the non-technical skills that support reliable team performance in high-risk clinical settings [3,24,26].

## 7. Limitations

This study has several limitations. The high baseline scores across measures introduce the possibility of a ceiling effect, limiting the ability to detect measurable improvements. The relatively short follow-up period (one month) may not capture longer-term impacts of the intervention. Additionally, the study was conducted within a single critical care context, with a small follow-up sample size, which may limit generalizability to other settings.

There was also attrition between baseline and follow-up, with 28 participants completing baseline surveys and 14 completing the one-month follow-up assessment. This limited the precision of the descriptive findings and may have introduced response bias. Specifically, participants who were more engaged with the program, had more positive experiences, or had greater capacity to complete follow-up surveys may have been more likely to respond. As a result, follow-up findings may overrepresent the perspectives of participants who were more satisfied with or engaged in TTrACE. In addition, baseline and follow-up responses could not be linked at the individual participant level, which prevented paired pre–post analyses and limited conclusions about individual-level change over time.

Although the ACE-15 and BPNSFS were previously published measures, the exploratory team-functioning items were assembled for the purposes of this pilot evaluation and were not validated as a single composite instrument. Psychometric evaluation of these exploratory domains was beyond the scope of this study and should be addressed in future work.

Because outcomes relied on self-reported perceptions rather than objective behavioural or patient-level measures, findings should be interpreted cautiously. Self-report data may be influenced by social desirability, recall bias, or participants’ overall perceptions of the program. The study did not include a control group, objective behavioural observations of teamwork, patient safety indicators, clinical outcomes, or workforce outcomes. As such, findings should be interpreted as descriptive and exploratory rather than causal.

## 8. Future Directions

Future research should explore the long-term sustainability of team functioning following training interventions and examine links to patient-level outcomes. Incorporating qualitative methods could provide deeper insight into how participants experience and apply team training in practice. Comparative studies across units with varying baseline levels of team functioning may also help clarify where such interventions have the greatest impact. Future research should also examine whether sustained investment in interprofessional team training contributes to workforce retention, burnout mitigation, and long-term patient safety outcomes across different ICU contexts.

## 9. Conclusions

In this pilot evaluation, baseline scores across collaboration, psychological safety, need satisfaction, and team functioning were high and remained stable at one-month follow-up, with descriptive improvements in perceived teamness and shared mental models. In already high-performing ICU environments, the value of structured interprofessional team training may lie less in producing large immediate measurable performance gains and more in sustaining the psychological, relational, and collaborative conditions required for consistently safe and effective care. TTrACE may therefore represent a useful approach to reinforcing high-functioning team environments in critical care, particularly when embedded as part of ongoing workforce development and team sustainment efforts.

## Figures and Tables

**Table 1:** Participant characteristics at baseline (*N* = 28).

Characteristic	*n* (%)
Profession	
Health disciplines	12 (43)
Nursing	9 (32)
Other	7 (25)
Years in profession	
9+ years	19 (68)
0–8 years	9 (32)
Years on Unit	
0–2 years	13 (46)
3–8 years	5 (18)
9+ years	10 (36)
Gender	
Female	23 (82)
Male	5 (18)
Age range (years)	
20–29	12 (43)
30–39	5 (18)
40–49	5 (18)
≥50	6 (21)
Highest education level	
College diploma/Undergraduate degree	17 (61)
Post-graduate degree	11 (39)

To protect participant confidentiality, categories with cell sizes < 5 were combined.

**Table 2:** Subscale scores of the Basic Psychological Need Satisfaction and Frustration Scale (BPNSFS) at baseline and 1-month follow-up.

BPNSFS Domain	Baseline (*n* = 27) Mean (SD)	Follow-Up (*n* = 13) Mean (SD)
Autonomy satisfaction	4.66 (0.65)	4.48 (0.53)
Competence satisfaction	5.17 (0.56)	5.21 (0.48)
Relatedness satisfaction	4.96 (0.51)	4.98 (0.44)
Autonomy frustration	3.05 (1.00)	3.10 (0.76)
Competence frustration	2.03 (0.90)	1.92 (0.61)
Relatedness frustration	1.83 (0.77)	1.92 (0.66)
Overall need satisfaction (mean of 3 satisfaction subscales)	4.93 (0.45)	4.89 (0.33)
Overall need frustration (mean of 3 frustration subscales)	2.30 (0.68)	2.31 (0.44)

**Table 3:** Exploratory team functioning domain scores at baseline and 1-month follow-up.

	Baseline (*n* = 27)	Post-Participation (*n* = 13)
Overall (20-item mean score)	5.27 (0.66)	5.56 (0.32)
Shared mental models (items 1–5)	5.28 (0.74)	5.91 (0.49)
Psychological safety & communication (items 6–12)	5.07 (1.05)	5.14 (0.61)
Perceived team effectiveness (items 13–20)	5.49 (0.74)	5.79 (0.52)

## Data Availability

The data presented in this study are available upon request from the corresponding author. The data are not publicly available due to privacy restrictions.
